# Relating Sensory, Cognitive, and Neural Factors to Older Persons' Perceptions about Happiness: An Exploratory Study

**DOI:** 10.1155/2018/4930385

**Published:** 2018-12-16

**Authors:** Alexandra J. Horne, Kimberly S. Chiew, Jie Zhuang, Linda K. George, R. Alison Adcock, Guy G. Potter, Eleonora M. Lad, Scott W. Cousins, Frank R. Lin, Sara K. Mamo, Nan-Kuei Chen, Abigail J. Maciejewski, Xuan Duong Fernandez, Heather E. Whitson

**Affiliations:** ^1^Duke University School of Medicine, Durham, NC, USA; ^2^Johns Hopkins University, Baltimore, MD, USA; ^3^Center for Cognitive Neuroscience, Duke University, Durham, NC, USA; ^4^Brain Imaging and Analysis Center, Duke University, Durham, NC, USA; ^5^Department of Sociology, Duke University, Durham, NC, USA; ^6^Center for the Study of Aging and Human Development, Duke University, Durham, NC, USA; ^7^Department of Psychiatry and Behavioral Sciences, Duke University School of Medicine, Durham, NC, USA; ^8^Department of Ophthalmology, Duke University School of Medicine, Durham, NC, USA; ^9^Department of Otolaryngology, Johns Hopkins University School of Medicine, Baltimore, MD, USA; ^10^Department of Communication Disorders, University of Massachusetts, Amherst, MA, USA; ^11^Geriatrics Research Education and Clinical Center, Durham VA Medical Center, Durham, NC, USA; ^12^Department of Medicine, Duke University School of Medicine, Durham, NC, USA

## Abstract

Despite increased rates of disease, disability, and social losses with aging, seniors consistently report higher levels of subjective well-being (SWB), a construct closely related to happiness, than younger adults. In this exploratory study, we utilized an available dataset to investigate how aspects of health commonly deteriorating with age, including sensory (i.e., vision and hearing) and cognitive status, relate to variability in self-described contributors to happiness. Community-dwelling seniors (*n* = 114) responded to a single-item prompt: “name things that make people happy.” 1731 responses were categorized into 13 domains of SWB via structured content analysis. Sensory health and cognition were assessed by Snellen visual acuity, pure-tone audiometry, and in-person administration of the Brief Test of Adult Cognition by Telephone (BTACT) battery. A subset of eligible participants (*n* = 57) underwent functional magnetic resonance imaging (fMRI) to assess resting state functional connectivity (FC) within a previously described dopaminergic network associated with reward processing. SWB response patterns were relatively stable across gender, sensory status, and cognitive performance with few exceptions. For example, hearing-impaired participants listed fewer determinants of SWB (13.59 vs. 17.16; *p* < 0.001) and were less likely to name things in the “special events” category. Participants with a higher proportion of responses in the “accomplishments” domain (e.g., winning, getting good grades) demonstrated increased FC between the ventral tegmental area and nucleus accumbens, regions implicated in reward and motivated behavior. While the framework for determinants of happiness among seniors was largely stable across the factors assessed here, our findings suggest that subtle changes in this construct may be linked to sensory loss. The possibility that perceptions about determinants of happiness might relate to differences in intrinsic connectivity within reward-related brain networks also warrants further investigation.

## 1. Introduction

Subjective well-being (SWB) refers to a subjective state encompassing people's longer-term levels of pleasant affect, lack of unpleasant affect, and life satisfaction [1]. Although well-being is a complex and multifaceted construct [2, 3], SWB can be generally understood as an index of happiness [4–6]. Research characterizing SWB over the lifespan has identified a paradox: although older adults experience declining physical health and increased personal losses (e.g., the deaths of spouse and friends), they report relatively stable or even increased levels of SWB [7, 8] and an increased ratio of positive to negative emotions relative to younger adults [9–11]. This “positivity effect” has been interpreted as partially reflecting a phenomenon termed *socioemotional selectivity*; that is, as individuals advance towards the end of their lifespan and perceive limitations in time, their goals shift such that they prioritize emotional satisfaction over goals that will pay off in the future (e.g., knowledge or power acquisition), thus promoting positive affect or greater happiness [12]. Furthermore, prior work suggests that the life domains (e.g., social relationships, wealth) that contribute to happiness may change with age; this may be due to changes in life situations, such as retirement or widowhood, or changes in health status [7].

Two aspects of health status that are prone to age-related decline and directly mediate one's lived experience are cognitive and sensory health. Mild cognitive impairment (MCI) is present in 3% to 19% of adults over the age of 65 years, with more than half of these individuals progressing to dementia over 5 years [13]. Compared to older adults with intact cognition, seniors with mild cognitive impairment tend to exhibit higher rates of loneliness and anxiety and increased difficulty in performing daily activities [14]. Likewise, impairments in vision and hearing may affect one's subjective state and become increasingly common in late life. The prevalence of uncorrectable vision impairment among Americans has been estimated as 16.1% among those aged 60–69 years and increases to 50% in Americans over age 80 years [15]. The prevalence of hearing loss among Americans over age 70 years has been estimated as 63.1% [16]. Multiple studies have demonstrated a relationship between vision and hearing impairments and lower quality of life, SWB, and other patient-centered outcomes [17–20].

Understanding how cognitive, vision, and hearing statuses relate to variability in self-described contributors to happiness may add to our understanding of age-related changes in SWB. One challenge to research in this area is that it can be difficult to study determinants of SWB in the setting of cognitive impairment, given inherent issues with measuring patient-reported variables in this population. In this exploratory analysis, we address these gaps with a novel approach to capture self-specified contributors to SWB, or happiness. We made use of data from a group of older adults with well-characterized sensory and cognitive status who had been asked to respond to the prompt, “Name things that make people happy.” While this task departs from multi-item scales traditionally used to characterize various dimensions of well-being [1, 7], the simple and open-ended nature of our measure allows feasible data collection among participants with varying degrees of age-related impairment and generates a diversity of responses that may reflect participants' own notions about determinants of happiness.

Next, in order to further explore relationships between self-described determinants of happiness and activity in a functional brain network of interest, we examined intrinsic, resting-state brain activity in a subset of our cohort that was eligible to undergo brain MRI. We were specifically interested in investigating resting-state activity within a functional network associated with reward anticipation and mesolimbic dopamine function [21], given the central role of dopamine in reward processing and motivated behavior [22, 23]. Our interest in relationships between perceived determinants of happiness and activity in this functional neural network was motivated by prior work that has suggested that individual variability in well-being might relate to brain function [24], particularly in regions associated with reward processing, such as the ventral striatum [25]. Although Heller et al. [25] characterized neural correlates of eudaimonic well-being, or one's sense of purpose, fulfillment, or meaning, as opposed to SWB, which is more typically linked to pleasure and happiness, we reasoned that pleasure is an integral component of reward [26]. Thus, we hypothesized that individual variation in perceived determinants of happiness might similarly scale with activity in reward-related brain regions. Exploring this relationship in older adults is novel and of interest, considering that age-related decline in dopaminergic activity has been well documented [27], just as the domains that contribute to SWB are also thought to shift with age [7, 28].

Overall, the present study seeks to advance our understanding of the complex relationships among sensory, cognitive, and neural health status and older adults' perceptions about determinants of SWB, or happiness. In an exploratory approach, we made use of qualitative data generated from a patient-centered and feasible task that asks individuals to “name things that make people happy.” A better understanding of how older adults' notions about happiness are linked to common, age-related impairments may shed light on SWB across the lifespan and is a necessary step toward our ultimate goal of designing programs and services to optimize happiness in seniors coping with insults to neural organs.

## 2. Methods

### 2.1. Study Population

Data were acquired from an ongoing study that examines cognitive and brain changes associated with age-related macular degeneration (AMD) (National Clinical Trial Registry #: NCT01996215). The parent study has enrolled 81 individuals with AMD as well as an age-balanced control group of 85 individuals with healthy eyes and normal vision. AMD is a condition that may affect one or both eyes and has a variable effect on visual acuity, depending on the stage and severity of the disease. The present analysis includes 114 community-dwelling individuals who had complete data on variables of interest, including neurocognitive assessment, eye examinations, audiology assessments, and health surveys. Participants were recruited from the Duke Eye Center clinics and Duke Aging Center registries. Exclusion criteria for the study were as follows: vision-limiting eye conditions other than AMD, diagnosis of moderate or severe dementia (per chart review or self-report from participant or family) or lack of capacity to provide consent (as assessed by trained study staff), and hearing loss severe enough to preclude participation in study interviews. Of the 114 participants in this analysis, 57 were willing and eligible to undergo functional MRI to characterize resting-state brain activity. Participants were excluded from the MRI portion of the study if they were left-handed, had history of brain surgery or brain pathology (e.g., brain tumor, history of stroke), significant claustrophobia, body weight >300 pounds, or had other contraindications to MRI (e.g., cardiac pacemakers, noncompatible implants).

### 2.2. Qualitative Data on SWB: Things that Make People Happy

We derived qualitative data about determinants of SWB from a single-item task that required participants to name, in a 60-second period, as many unique responses as possible within the category: “things that make people happy”. Neuropsychological assessments were administered by trained study personnel under standardized conditions. The “happy task” was administered as part of the Fuld Object Memory Evaluation [29], in which the task is used to distract participants between memory trials. The assessments were audio-recorded and individual responses were later transcribed into the study database.

The present investigation was conducted on an exploratory basis because, within the data of the parent study, we noted striking heterogeneity in the content of responses generated by the “happy task.” We hypothesized that the nature of responses may provide a unique window on individual differences in perceptions about SWB within an older-adult population with varying degrees of sensory and cognitive impairment.

### 2.3. Vision and Hearing Impairment

Visual acuity was measured in the Duke Eye Center Clinical Research Unit. Vision impairment was defined as Snellen best-corrected visual acuity of 20/40 or less in the better eye. We chose this definition because, in people with central vision loss from AMD, quality of life has been more closely linked to vision in the better seeing eye (as opposed to the worse seeing eye) [30]. Audiometry was assessed with an Interacoustics AD629 Audiometer with ER3A insert earphones in a sound-attenuating booth. Hearing loss was defined as a pur-tone average greater than 25 dB HL in the better ear, averaged over frequencies of 0.5, 1, 2, and 4 kHz, which is a standard definition of function-limiting hearing impairment [16].

### 2.4. Cognitive Measures

The parent study assessed global cognitive performance with the Brief Test of Adult Cognition by Telephone (BTACT), a validated cognitive battery that evaluates episodic verbal memory, working memory span, verbal fluency, inductive reasoning, and speed of processing [31]. Because it was designed for administration by telephone, the BTACT does not include any items or tasks that rely on vision, which was ideal in this patient population. The BTACT was administered to participants in person by a trained individual in a quiet exam room. In the present study, global cognitive status was quantified via a composite score derived from BTACT performance data via factor analysis. To assign a composite score to each individual, a principal axis factor was performed on the six BTACT measures, which suggested a single-factor solution (one eigenvalue > 1), consistent with the previous literature [31]. The factor matrix showed moderate to strong loadings for all six tests, ranging from 0.37 to 0.70.

One of the six tests in the BTACT is a semantic verbal fluency task: the Animal Naming task, which requires participants to name as many types of animals as they can in 60 seconds [32]. The total number of responses generated by the “happy task” is also a reflection of verbal fluency. Thus, in analyses that considered total number of responses to the “happy task,” we used scores on the Animal Naming task as a covariable to adjust for semantic fluency ability.

### 2.5. Health and Demographic Variables

Participant age was recorded from the medical record. Gender and education level were collected by self-report. Depression screening was performed via verbal administration of the 15-question Geriatric Depression Scale (GDS) [33]. A comorbidity count was created based on the number of affirmative response to a question that asked: “Has a doctor ever told you that you have any of the following health conditions: coronary artery disease, congestive heart failure, heart arrhythmias, arthritis, COPD, stroke/TIAs, diabetes, hypertension, liver disease, kidney disease, cancer, dementia or Alzheimer's disease, Parkinson's disease, osteoporosis, ulcers in the stomach or duodenum, urinary or stool incontinence, and/or insomnia?”

### 2.6. MRI Data Acquisition

Structural and functional MRI data were collected on a 3T GE MR750 scanner at the Duke/UNC Brain Imaging and Analysis Center (BIAC). An eight-channel head coil was used for radio frequency (RF) reception (General Election, Milwaukee Wisconsin, USA). Sagittal T1-weighted localizer images were acquired and used to define a volume for data collection and high-order shimming. High-resolution structural images were acquired using a 3D fSPGR pulse sequence (TR = 8.156 ms; TE = 3.18 ms; TI = 450 ms; FOV = 25.6 cm^2^; flip angle = 12°; voxel size = 1 × 1 × 1 mm; 166 contiguous slices, sense factor = 2). A semiautomated high-order shimming program was used to ensure global field homogeneity. T2^*∗*^-weighted functional images were acquired during resting state, using a gradient echo-planar sequence sensitive to BOLD contrast (TR = 2 s; TE = 27 ms; flip angle 77°; FOV = 24 cm^2^; SENSE factor = 1; voxel size = 3.75 × 3.75 × 4 mm; 34 contiguous oblique axial slices, parallel to the anterior commissure-posterior commissure line, interleaved acquisition) in two 180-volume (∼6 minute) runs. In each of these two runs, four initial radio-frequency (RF) excitations were performed to achieve steady-state equilibrium and were subsequently discarded. During these resting state runs, participants were instructed to “stay awake and think about nothing in particular”. Due to the varying visual abilities in this cohort, all participants were instructed to close their eyes during imaging acquisition. Participants remained awake for the duration of MRI data acquisition, which was confirmed by verbal communication between runs.

### 2.7. MRI Preprocessing

Preprocessing was conducted through Duke/UNC BIAC processing daemons based on the tools from the Oxford Centre for Functional MRI of the Brain's Software Library (FSL version 4.0, https://www.fmrib.ox.ac.uk/fsl). The first four images were removed from each resting state run to achieve steady-state equilibrium. All remaining functional images were aligned with respect to the first image within each run to correct for head movements during data acquisition. A bandpass filter was applied to filter the functional data in the time dimension so that frequencies were retained between 0.001 Hz and 0.08 Hz. The aligned and filtered images were spatially normalized to Montreal Neurological Institute (MNI) space during a 12-degree-of-freedom affine transformation implemented in FSL's Linear Image Registration Tool (FLIRT). We further removed constant offsets and linear drift over each run and regressed out the six rigid body head motion parameters, the signal averaged over the white matter, and the signal averaged over the cerebrospinal fluid regions to reduce nonneuronal influence to BOLD corrections. The normalized images were smoothed using an isotropic Gaussian kernel of 5 mm full-width-half-maximum. The resulting voxel size was 3.75 × 3.75 × 4 mm.

### 2.8. Data Analysis

#### 2.8.1. Qualitative Analysis of SWB Data

A qualitative content analysis [34] was performed to characterize differences in responses to the “happy task.” As a first step, two independent reviewers categorized the responses into 10 mutually exclusive domains. The original framework of 10 domains was based on seven determinants of SWB that have been well described in the literature (social relationships, financial/material wealth, health factors, religious involvement, volunteerism/altruism, exercise and physical activity, and accomplishments) [35], plus three domains that were observed to be recurring categories in our data: avocation (e.g., hobbies), ingestibles (e.g., food and beverages), and special events (birthdays and weddings). Next, the two reviewers met to compare categorizations and revise the framework. An eleventh domain (“nature”) was created to capture nature-related responses as an emergent theme identified independently by both reviewers, for which responses did not neatly fit into the initial categories. The nature domain included responses such as “mountains” and “sunsets.” Some decision rules were established to guide categorization (e.g., include pets in the social relationship category). Responses were recategorized as needed based on the revised framework and decision rules. Finally, a committee of three coauthors met to adjudicate any remaining disparate categorizations and discuss all responses that had not been assigned to a category. At this meeting, a twelfth domain (“positive emotion”) was added to our framework to capture responses such as “smiles” and “laughter” that were felt to represent a small but recurrent theme. Additional decision rules were agreed upon to resolve discordant categorizations (e.g., if the response used active voice for a sporting activity (e.g., “playing basketball”), it was categorized as “exercise/physical activity”, whereas passive references to sports (e.g., “basketball”), for which it could not be determined whether the enjoyment was derived from playing or spectating, were categorized as avocation/hobby). This iterative process produced almost complete theme saturation, with 99.08% of responses (1715/1731) categorized into one of 12 mutually exclusive domains (as noted above: social relationships, financial/material wealth, health factors, religious involvement, volunteerism/altruism, exercise and physical activity, accomplishments, avocation/hobbies, ingestibles, special events, nature, and positive emotion).

Next, to explore potential contributions of sensory health to individual variability in perceived determinants of happiness in this sample, we created a secondary category of “vision-dependent” responses. All responses that were determined by reviewer consensus to rely heavily on visual ability (e.g., working crossword puzzles, watching television, driving a vehicle, and reading) were assigned to this category, regardless of their primary assignment in one of the 12 mutually exclusive categories.

#### 2.8.2. MRI Data Analysis

**Figure 1:**
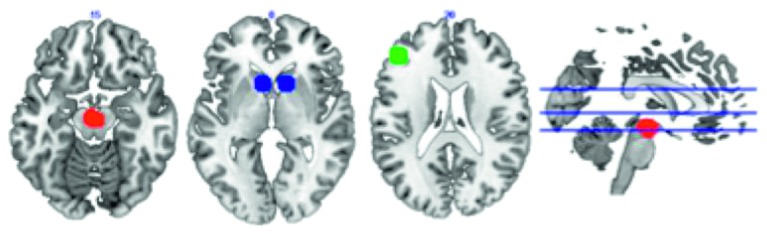
Map demonstrating the selected mesocorticolimbic structures in the dopaminergic reward network, using peak coordinates described by Ballard et al. [21]. Note: red = ventral tegmental area (VTA), blue = nucleus accumbens (NAcc), green = dorsolateral prefrontal cortex (dlPFC).

The BOLD signal, measured via fMRI, is used as a marker of region-specific metabolic activity within the brain. Regions whose BOLD signal fluctuations show a high degree of temporal correlation are presumed to constitute a tightly coupled neural network and are said to be functionally connected [36]. Here, we estimated the functional connectivity (FC) within selected regions associated with reward anticipation within the dopaminergic mesolimbic system (reward network; i.e., ventral tegmental area, nucleus accumbens, and dorsolateral prefrontal cortex) [21]. Regions of interest (ROIs) were drawn as 8 mm spheres centered on peak coordinates identified via prior work [21] and were converted to MNI space. These ROIs are displayed in Figure 1. To quantify FC, participants' BOLD time series data were averaged within each selected ROI and Pearson correlation coefficients were calculated to examine relationships between average levels of BOLD activity in pairs of regions within the network.

### 2.9. Statistical Analyses

Descriptive statistics were used to characterize the sample. For each participant, we calculated the total number of responses to the “happy task” as well as the proportion of responses belonging to each of the 12 mutually exclusive domains as well as the “vision-dependent” task domain. Additionally, we created a dichotomous variable for each of the 12 SWB domains that indicated whether or not the participant gave any response within that domain.

#### 2.9.1. SWB/Happiness Responses and Gender, Vision, and Hearing Status

Two-tailed *t*-tests were used to compare the mean proportion of responses in each domain category, across three dichotomous groups: gender (male vs female), hearing status (unimpaired vs impaired), and vision status (unimpaired vs impaired). We used Pearson's chi-squared tests to compare the proportions of individuals in each of these dichotomous groupings who listed at least one response in each domain.

#### 2.9.2. SWB/Happiness Responses and Cognitive Performance

Global cognitive performance, indexed via BTACT composite score (derived via factor analysis on our sample of data), exhibited a Gaussian distribution and was analyzed as a continuous variable. We do not dichotomize the cognitive variable because we lack the data to determine a valid cut-point to delineate normal cognition based on this composite score. Pearson correlations assessed the relationship between cognitive performance and total number of “happy task” responses, as well as the proportion of responses in each of the 13 domains.

#### 2.9.3. Adjusted Models

After examining the bivariate relationships between SWB response patterns and gender, vision status, and hearing status, fully adjusted regression models were built. All models included the following independent variables included as predictors: age, gender, education level, GDS score, number of comorbid medical conditions, hearing status (unimpaired or impaired), and vision status (unimpaired or impaired). We constructed adjusted models with the following dependent variables: total number of SWB responses, proportion of responses in any domain with a mean proportion ≥20%. We did not examine adjusted models of proportions if the mean proportion was <20% because in such cases, the distribution of proportions is highly skewed, with many participants having zero responses in that domain. In models where the dependent variable was the total number of SWB responses, Animal Naming score was also included as an independent variable, to control for verbal fluency ability. In models where the dependent variable was proportion of responses in a given domain, BTACT composite score was included as an independent variable to adjust for global cognitive status. We did not include both Animal Naming score and BTACT composite score in the same models, as these two cognitive test scores were highly collinear. Collinearity of the other variables in the models was evaluated, and no two variables exceeded our threshold for unacceptable collinearity, which was a Pearson's correlation coefficient (CC) >0.7 (the highest CC was 0.49 for hearing impairment and age) (Table 1).

#### 2.9.4. SWB/Happiness Responses and Brain Connectivity

To examine relationships between the SWB data and reward network activity, average FC between pairs of selected regions in this network, as previously described [21], was calculated. The regions in the previously described network included the ventral tegmental area (VTA) (left and right VTA averaged together), nucleus accumbens (NAcc) (left and right NAcc averaged together), and left dorsolateral prefrontal cortex (DLPFC). We examined connectivity between each pairwise combination of nodes (VTA-NAcc; VTA-DLPFC; NAcc-DLPFC). FC between each pair of regions was subsequently correlated to the proportion of responses belonging to each of the 12 domains. Fully adjusted regression models were built with FC (of each region pairing) as the dependent variable and the following independent variables: proportion of responses in a domain, age, gender, education, GDS score, number of comorbidities, vision status (impaired/not impaired), hearing status (impaired/not impaired), and BTACT composite score.

Statistical analyses were performed using JMP software from SAS (Cary, NC). Due to multiple comparisons performed in this exploratory analysis, to reduce the risk of alpha error accounting for spurious conclusions, we set our *p* value threshold for significance at 0.01, which is more conservative than the traditional level of 0.05.

## 3. Results

### 3.1. Participant Characteristics

Participant demographics and clinical characteristics are summarized in Table 2. Mean age of participants was 74.6 years with 54.4% females. Mean short-form GDS score was 1.4 (out of 15) and only three of 114 participants scored within ranges suggestive of depression (i.e., GDS score > 5). While 52.6% of our study samples are diagnosed with AMD in at least one eye, only 21.9% meet our definition of function-limiting vision impairment, which is based on visual acuity in the better-seeing eye. Some AMD participants had unilateral AMD and/or relatively preserved visual acuity despite AMD. Participants with bilateral hearing impairment represented 55.3% of the cohort. Seventeen participants had both vision and hearing impairments, meaning that dual sensory impairment affected 14.9% of the cohort.

With respect to most characteristics, the 57 participants who received an fMRI were similar to those who did not, although those who were eligible and willing to under fMRI tended to report fewer chronic conditions (fMRI subset *M* = 2.2, *SD* = 1.5; all participants *M* = 2.9, *SD* = 2.1).

### 3.2. Determinants of SWB: “Happy Task” Response Patterns

The mean number of responses to the “happy task” was 15.2 (SD: 5.5, range 4–38). Of the 1731 responses across all participants, 1715 (99.08%) were categorized into one of 12 mutually exclusive domains of SWB, as described in the Methods section. 237 (13.7%) of the 1731 total responses to the SWB task were additionally categorized in the “vision-dependent” category. The results of the qualitative content analysis are summarized in Table 3. Responses most frequently belonged to the domains of social relationships and avocation/hobbies. Other highly prevalent domains included financial/material wealth, ingestibles (i.e., food, beverage), and nature-themed responses.

### 3.3. Response Patterns According to Gender, Vision Status, and Hearing Status

There was no significant difference in the number of responses to the “happy task” by gender (male: *M* = 14.27, *SD* = 4.79; female: *M* = 15.95, *SD* = 5.99; *t*(112) = 1.67, *p* = 0.11). As shown in Table 4, no gender-based differences in response patterns met our criteria for statistical significance (*p* < 0.01), although males were somewhat more likely to list responses in categories of ingestibles (73.08% vs. 54.84%, *p*=0.04) and financial wealth (65.38% vs. 48.39%, *p*=0.07), while females were more likely to list social relationship responses (96.77% vs. 86.54%, *p*=0.04). Individuals with and without vision impairment listed similar numbers of responses to the SWB prompt (vision-impaired: *M* = 14.52, *SD* = 4.82; non-vision-impaired: *M* = 15.37, *SD* = 5.71; *t*(45) = 0.75, *p*=0.50). Visually impaired participants were more likely to express avocation (100% vs. 84.27%, *p*=0.03) and vision-dependent (92.00% vs. 69.66%, *p*=0.02) responses (e.g., completing a crossword puzzle), compared to nonvisually impaired peers, although these differences do not meet our threshold for significance. Participants with hearing impairment listed significantly fewer responses overall (hearing-impaired: *M* = 13.59, *SD* = 4.72; non-hearing-impaired: *M* = 17.16, *SD* = 5.83; *t*(96) = 3.53, *p*=0.001), although this result did not retain significance after adjustment for age, gender, education, GDS score, Animal Naming score, number of comorbid medical conditions, and vision impairment status (*p*=0.06; see Table 5). Also, adjusted analyses (Table 5) revealed that hearing-impaired participants listed higher proportions of social relationship responses (*t*(111) = 3.22, *p*=0.01) and in unadjusted analyses (Table 4), lower proportions of special events responses (e.g., holidays and parties, *t*(85) = 2.51, *p*=0.01), compared to non-hearing-impaired peers.

### 3.4. Response Patterns and Cognitive Status

As expected, number of responses to the “happy task” was associated with both Animal Naming task verbal fluency scores (*R*^2^ (112) = 0.28, *p*=0.001) and BTACT composite scores (*R*^2^ (112) = 0.23, *p*=0.001). As BTACT composite score declined, the proportion of responses belonging to the social relationships category increased (*R*^2^ (112) = 0.06, *p*=0.01). No significant relationships were observed between cognitive status and proportion of responses in other SWB domains.

### 3.5. Response Patterns and Brain Connectivity in the Mesolimbic Dopaminergic Reward Network

As summarized in Table 6, participants who listed an accomplishment-themed response (e.g., getting a promotion, earning an “A” on an exam, winning a prize) had significantly higher mean resting FC between VTA and NAcc, compared to those who did not list an accomplishment-themed response (*M* = 0.624, *SD* = 0.182 vs. *M* = 0.477, *SD* = 0.200; *t*(55) = 2.58, *p*=0.01). In addition, as the proportion of responses belonging to the accomplishment domain increased, mean connectivity between VTA and NAcc increased (*R*^2^ (55) = 0.104, *p*=0.01) and remained significant in fully adjusted multivariate regression models. Participants with higher GDS scores tended to have lower mean functional connectivity between VTA and NAcc (*R*^2^ (55) = 0.09, *p*=0.02); however, this relationship was attenuated in the fully adjusted model.

## 4. Discussion

This exploratory study investigates how interperson variability in self-expressed determinants of happiness, generated by the simple task “name things that make people happy,” relates to sensory and cognitive health in a group of older adults. We additionally investigated relationships between response patterns and neural activity in a prespecified brain network. To our knowledge, this is the first study to examine potential neural underpinnings of SWB determinants among older adults with varying levels of age-related sensory and cognitive function.

Qualitative content analysis and subsequent statistical analyses revealed few significant differences in response patterns based on participants' gender, vision, hearing, or cognitive status. Hearing-impaired individuals listed fewer responses overall, but this difference did not remain statistically significant after adjusting for demographic variables, depressive symptoms, comorbidity, vision status, and semantic fluency score (Animal Naming). The proportion of responses categorized into 13 different “domains” of SWB was also fairly consistent across the participant groups. The fact that a relatively stable pattern of responses was elicited in this cohort, in men and women and across varying degrees of sensory and cognitive function, supports the validity of existing SWB frameworks and important commonalities in perceptions of happiness.

A few group differences in response patterns did exceed our conservative threshold for statistical significance and are notable. Worse global cognitive scores were significantly associated with higher proportions of responses in the social relationship domain, which was also the most common domain overall. Hearing-impaired individuals also listed higher proportions of social relationship responses and lower proportions of special event responses, compared to individuals with unimpaired hearing. The reasons for these patterns cannot be determined from available data, and there is a paucity of literature on how changes in cognition and sensory function may influence the drivers of one's happiness. One possibility is that impaired individuals are more dependent on friends and family, and thus the role of social relationships in determining happiness becomes especially salient in the setting of cognitive and sensory impairment. An alternative possibility is that hearing or cognitive impairments may challenge social relationships, creating a tendency for impaired individuals to be more aware of them. This rationale may be consistent with the separate (but not statistically significant) finding that vision-impaired individuals were more likely to list responses in the category of vision-dependent activities (e.g., painting and working crossword puzzles). A tendency to list more of these activities as “things that make people happy” may reflect nostalgic thoughts, or greater appreciation for activities that have been rendered more difficult (or impossible) by one's sensory loss. On the contrary, the tendency for hearing-impaired individuals to exhibit a lower proportion of responses about special events (e.g., weddings, Christmas parties) may reflect reduced enjoyment of such events due to hearing impairment.

In light of the high prevalence of sensory and cognitive impairment in the aging population, further research to understand how these aspects of function shape one's happiness is merited. Our study was motivated by previous work which has shown that individuals with sensory and cognitive deficits tend to report diminished quality of life and life satisfaction [37–40]. By identifying differences in perceived contributors to happiness, we hoped the present study would hint at which domains of SBW are most threatened by age-related impairments, or may suggest opportunities to preserve quality of life for impaired individuals. Overall, our findings point toward a relatively stable framework of determinants of happiness, but suggest that interventions that bolster social relationships may be especially valuable for people with cognitive and hearing impairments.

We also examined relationships between self-described determinants of happiness and average connectivity levels within a functional brain network. We observed that functional connectivity between the VTA and NAcc was higher in those who listed accomplishment-themed responses, versus those who listed fewer or no responses in that domain. Responses in the accomplishment domain included items such as “winning” or “getting promoted”, and it is possible that individuals who listed responses in that domain were primed, at a neural level, to associate feelings of happiness or pleasure with reward pursuit.

Primate studies have identified structural connectivity between the VTA and both the NAcc and PFC [41]. Importantly, the network we examined was identified in the context of a reward anticipation task [21]. A more recent human study examined resting-state connectivity of dopaminergic midbrain regions and observed intrinsic coupling between the VTA and NAcc, but not the VTA and PFC, at rest [42]. The authors suggest that VTA-PFC coupling might be observed specifically during task engagement or initiation of motivated behavior (hence observation of VTA-PFC connectivity during task performance in Ballard et al. [21]), but not during rest. Additionally, animal studies have shown that the VTA sends dopaminergic projections to the NAcc [43, 44] and the NAcc sends back modulatory input to VTA [45]. In general, these reciprocal communications are thought to be an integral component of the mesolimbic dopamine circuit mediating reward and motivation—a circuit which might include PFC during task engagement, but potentially not while in the resting state. If this is the case, our observation that participants with more reward-themed responses did not exhibit higher connectivity between VTA-PFC or NAcc-PFC at rest is not unexpected, although these individuals might exhibit stronger connections between these regions during task performance. Testing this prediction remains a direction for future research.

Another novel aspect of our analysis was the use of qualitative data from the patient-centered SWB task, which is based on unique lists generated by our participants. The tool is feasible, even in people with some degree of cognitive and hearing impairments, and it provides a rich diversity in the types of responses generated. In addition, the open-ended nature of the prompt allows for spontaneous insights about perceived contributors to happiness, rather than eliciting SWB data through any particular theoretical lens. In addition, because participants are asked to name things that make people happy (rather than “make *you* happy”), the tool may reveal perceptions about societal expectations of happiness, as opposed to more personal determinants of happiness. The pattern of responses elicited is consistent with prior literature which suggests that global life satisfaction, or personal happiness, might be characterized as a multidimensional construct with many domain-specific determinants [7].

Our study has important limitations. The “happy task” we used to gain insights about determinants of SWB has not been validated for this purpose, and this approach should be considered exploratory. If participants were asked about determinants of their own happiness (rather than “name things that make people happy”), we may have observed greater differences in response patterns as a function of sensory and cognitive status. Additionally, we cannot infer from the data how the specific SWB categories we identified relate to the respondents' concept of happiness (e.g., did respondents perceive happiness as a multidimensional construct, or a unidimensional construct with multiple potential predictors?). Another limitation is that although the task is feasible in people with some cognitive impairment, certain cognitive deficits likely influence performance on the task. The parent study excluded individuals with a known diagnosis of dementia or who lacked capacity to give consent, but we did observe variations in cognitive test performance within the study population. Although we attempted to adjust for cognitive ability statistically, formal testing for dementia-related diagnoses was not performed. Despite these limitations, we believe the tool has face validity as a means to reveal individual perspectives about factors that contribute to subjective sense of happiness.

Additional limitations of the study include its cross-sectional design, which prohibits us from inferring causal attributions from the observed correlations. Despite attempts to control for a number of confounders, including demographics and health factors, it remains possible that additional confounding factors influenced the relationships and group differences described here. Further, this study examined the effect of visual, hearing, and cognitive impairment separately, but future studies should investigate how multiple, coexisting impairments might interact to impact well-being. Lastly, given the exploratory nature of our investigation, analysis was conducted without correction for multiple comparisons, although we applied a conservative statistical significance threshold. Given that this particular single-item measure is unique to the present study, we felt that the use of an exploratory approach was justified in characterizing the types of response patterns generated in different populations; however, these findings require confirmation, as well as characterization of test-retest reliability, in a follow-up sample.

In conclusion, in an older adult sample, we found that sensory and cognitive statuses were associated with relatively few differences in the types and patterns of responses elicited by a simple prompt: “name things that make people happy”. The most notable exception was that individuals with hearing impairment produced a higher proportion of responses related to “social relationships” and a lower proportion of responses related to “special events”. Although it is perhaps intuitive that subjective perceptions about determinants of happiness and well-being might be influenced by our ability to perceive the environment, interact with people around us, and make sense of those inputs and experiences, this study identified a specific relationship between an age-related sensory deficit and determinants of SWB. In addition, we found that individual differences in intrinsic functional connectivity within a brain network associated with reward processing may be associated with differences in perceived contributors to happiness. These results are exploratory, but were motivated by prior work indicating relationships between neural function and individual variability in well-being [24, 25]. Future longitudinal studies, qualitative research, and task-based MRI research will help elucidate further how age-related health changes may affect SWB, and how differences in our notions about happiness may be supported by neural function, with the ultimate goal of optimizing programs and services that promote SWB in seniors coping with sensory and cognitive loss.

## Figures and Tables

**Table 1:** Correlation coefficient matrix of variables included in adjusted models.

	Age	Gender	Education	GDS	CC	VI	HI	BTACT
Age	1.0	−0.07	−0.01	−0.04	0.14	0.13	0.49^*∗∗∗*^	−0.29^*∗∗*^
Gender		1.0	−0.07	0.03	−0.15	0.10	−0.19^*∗*^	0.08
Education			1.0	−0.14	−0.30^*∗∗*^	0.10	−0.19^*∗*^	0.36^*∗∗∗*^
GDS				1.0	0.48^*∗∗∗*^	0.07	0.08	−0.10
CC					1.0	−0.07	0.20^*∗*^	−0.26^*∗∗*^
VI						1.0	0.14	−0.16
HI							1.0	−0.27^*∗∗*^
BTACT								1.0

GDS, geriatric depression screen score; CC, chronic condition count; VI, vision impairment; HI, hearing impairment; BTACT, Brief Test of Adult Cognition by Telephone. ^*∗*^*p* < 0.05, ^*∗∗*^*p* < 0.01, ^*∗∗∗*^*p* < 0.001.

**Table 2:** Participant demographics and clinical characteristics.

Characteristics	All participants (*n* = 114)	Subset with fMRI (*n* = 57)
Age, mean (SD), y	74.6 (7.8)	73.4 (8.1)
Women, %	54.4	64.9
College degree or higher, %	57.9	61.4
Chronic medical conditions, mean (SD)	2.9 (2.1)	2.2 (1.5)^d^
Geriatric depression scale (GDS), mean (SD)^a^	1.4 (1.9)	1.2 (1.6)
Vision-impaired, %^b^	21.9	24.6
Hearing impaired, %^c^	55.3	49.1

^a^Geriatric depression scale (GDS) short-form: 15-item screening tool used to identify depression in older adults. ^b^Defined as visual acuity 20/40 or less in the better eye. ^c^Defined as pure-tone average >25 dB HL in the better ear averaged over frequencies of 0.5, 1, 2, and 4 kHz. ^d^fMRI subset significantly differs from participants without fMRI (at *p* < 0.05 level).

**Table 3:** Qualitative content analysis of responses to the task “name things that make people happy” (*n* = 114 participants, *n* = 1731 responses).

Domains	% participants with at least one response in the corresponding domain (%)	Mean proportion of responses per participant
Social relationships^a^	92.11	0.23
Material wealth^b^	56.14	0.08
Physical activity^c^	47.37	0.06
Accomplishments^d^	33.33	0.04
Religious involvement^e^	19.30	0.01
Health factors^f^	15.79	0.02
Volunteerism/altruism^g^	12.28	0.02
Avocation/hobbies^h^	87.72	0.28
Ingestibles^i^	63.16	0.09
Special events^j^	37.72	0.06
Nature^k^	50.88	0.08
Positive emotion^l^	31.58	0.03
Vision-dependent tasks^m^	74.56	0.14

^a^Examples include marriage, love, spouse, family, children, companionship, and friends. ^b^Examples include money, new house, nice clothes, and jewelry. ^c^Examples include going to the gym, exercise, running, swimming, and playing sports. ^d^Examples include getting a promotion, earning a good grade, succeeding, and winning a prize. ^e^Examples include prayer, fellowship, attending church, and faith. ^f^Examples include good health, sight, dressing oneself, and hearing. ^g^Examples include volunteer work, being helpful, visiting the sick, and doing favors for others. ^h^Examples include watching sports, playing cards, reading, gardening, and fishing. ^i^Examples include eating, specific food items, wine, and beer. ^j^Examples include holidays, birthdays, parties, graduations, and weddings. ^k^Examples include scenery, sunshine, ocean, mountains, and nice weather. ^l^Examples include laughter, smiles, fun, jokes, and happiness. ^m^Examples include completing a crossword puzzle, watching television, and driving a vehicle.

**Table 4:** Name things that make people happy: comparison of response patterns by gender, vision, and hearing status.

Domains of SWB	Gender			Vision			Hearing		
	Male	Female	*p* ^a^	Nml	Abn	*p* ^a^	Nml	Abn	*p* ^a^
Social relationships	86.54	96.77	0.04	89.89	100.0	0.10	86.27	96.83	0.04
Material wealth	65.38	48.39	0.07	59.55	44.00	0.17	56.86	55.56	0.89
Physical activity	40.38	53.23	0.17	44.94	56.00	0.33	54.90	41.27	0.15
Accomplishments	32.69	33.87	0.89	37.08	20.00	0.11	31.37	34.92	0.69
Religion	21.15	17.74	0.21	19.10	20.00	0.92	15.69	22.22	0.38
Health factors	15.38	16.13	0.91	15.73	16.00	0.97	15.69	15.87	0.98
Volunteerism/altruism	7.69	16.13	0.17	11.24	16.00	0.52	7.84	15.87	0.19
Avocation/hobbies	84.62	90.32	0.36	84.27	100.0	0.03	92.16	84.13	0.19
Ingestibles	73.08	54.84	0.04	64.04	60.00	0.71	66.67	60.32	0.48
Special events	32.69	41.94	0.31	40.45	28.00	0.26	50.98	26.98	<0.01
Nature	44.23	56.45	0.19	52.81	44.00	0.44	58.82	44.44	0.13
Positive emotion	34.62	29.03	0.52	28.09	44.00	0.13	35.29	28.57	0.44
Vision-dependent tasks	69.23	79.03	0.23	69.66	92.00	0.02	80.39	69.84	0.20

SWB, subjective well-being; Nml, normal; Abn, abnormal. ^a^*p* values for the comparison of the proportion of individuals in each group who listed any response in that domain (by two-tailed *t*-test). Due to multiple comparisons in this exploratory analysis, our alpha error threshold for statistical significance was set at *p* ≤ 0.01.

**Table 5:** Predictors of response patterns: results of multivariate regression models.

Model variables	“Happy” task score^a^		Proportion of responses belonging to the “social relationship” domain		Proportion of responses belonging to the “avocation/hobbies” domain	
	*β*	*P*	*β*	*p*	*β*	*p*
Age	−0.01	0.93	−0.20	0.05	0.00	0.08
Gender	0.97	0.29	0.10	0.28	0.06	0.12
Education level	0.38	0.37	0.08			0.89
< High school	—	—	0.18	—	−0.02	—
High school	—	—	0.17	—	−0.04	—
College	—	—	−0.35	—	−0.14	—
Graduate	—	—	0.08	—	−0.01	—
GDS-SF score	−0.13	0.61	0.05	0.63	0.00	0.69
Comorbid medical conditions	−0.26	0.27	−0.05	0.65	0.01	0.26
BTACT composite score	N/A	N/A	−0.17	0.09	0.03	0.23
Animal Naming score	0.45	<0.001	N/A	N/A	N/A	N/A
Hearing status	−2.00	0.06	−0.39	<0.001	−0.01	0.76
Vision status	−0.65	0.55	−0.03	0.71	0.09	0.04

Note. In the model that predicted SWB “happy” task score, Animal Naming score was included to control for intrinsic semantic fluency ability; in the model that predicted the proportion of responses (not total number of responses), we adjusted instead for global cognitive BTACT score. GDS-SF, geriatric depression scale short-form; BTACT, Brief Test of Adult Cognition by Telephone. ^a^Total number of responses to the 60-second “name things that make people happy” task.

**Table 6:** Resting-state functional connectivity of mesocorticolimbic regions in a reward network, among participants with and without “Happy task” responses in the accomplishment domain (*n* = 57).

Regions of interest	Function connectivity, mean		*p* ^a^
	+ Accomplishment	− Accomplishment	
VTA – NAcc	0.63	0.48	0.01
VTA – dlPFC	−0.07	−0.03	0.61
NAcc – dlPFC	−0.15	−0.04	0.20

VTA, ventral tegmental area; NAcc, nucleus accumbens; dlPFC, dorsolateral prefrontal cortex. ^a^*p* values derived from regression models in which dependent variable is functional connectivity (FC). Adjusted models included the following independent variables: proportion of responses in accomplishment domain, age, gender, education level, depression score, comorbidity count, BTACT cognitive score, vision status (impaired/unimpaired), hearing status (impaired/unimpaired). The *p* value indicates whether there was a significant association between FC and proportion of responses in the accomplishment domain.

## Data Availability

Data may be made available upon request to Dr. Heather Whitson: heather.whitson@duke.edu. Requests are subject to privacy and regulatory stipulations of the United States of America and Duke University Institutional Review Board.
